# Air Quality Forecast by Statistical Methods: Application to Portugal and Macao

**DOI:** 10.3389/fdata.2022.826517

**Published:** 2022-03-10

**Authors:** Luísa Mendes, Joana Monjardino, Francisco Ferreira

**Affiliations:** ^1^Department of Environmental Sciences and Engineering, NOVA School of Sciences and Technology, NOVA University Lisbon, Lisbon, Portugal; ^2^Center for Environmental and Sustainability Research, NOVA School of Sciences and Technology, NOVA University Lisbon, Lisbon, Portugal

**Keywords:** particulate matter, ozone, nitrogen dioxide, air quality, classification and regression trees, multiple regression

## Abstract

Air pollution is a major concern issue for most countries in the world. In Portugal and Macao, the values of nitrogen dioxide (NO_2_), particulate matter (PM) and ozone (O_3_) are frequently above the concentration thresholds accepted as “good air quality.” Portugal follows the European Union (EU) legislation (Directive 2008/50/EC) on air quality and Macao the air quality guidelines (AQG) from the WHO. Air quality forecasts are very important mitigation tools because of their ability to anticipate pollution events, and issue early warnings, allowing to take preventive measures and reduce impacts, by avoiding exposure. The work presented here refers to the statistical forecast of air pollutants for three regions: Greater Lisbon Area, Madeira Autonomous Region (both located in Portugal), and Macao Special Administrative Region (in Southern China). The presented statistical approach combines Classification and Regression Tree (CART) and multiple regression (MR) analysis to obtain optimized regression models. This consolidated methodology is now in operation for more than a decade in Portugal, and is subject to regular updates that reflect the ongoing research and the changes in the air quality monitoring network. Recently, the same methodology was applied to Macao in collaboration with the Macao Meteorological and Geophysical Bureau (SMG). Here, a statistical approach for air quality forecasting is described that has been proven to be successful, being able to forecast PM_10_, PM_2.5_, NO_2_, and O_3_ concentrations, for the next day, with a good performance. In general, all the models have shown a good agreement between the observed and forecasted concentrations (with *R*^2^ from 0.50 to 0.89), and were able to follow the concentration evolution trend. For some cases, there is a slight delay in the prediction trend. Moreover, the results obtained for pollution episodes have proven that statistical forecast can be an effective way of protecting public health.

## Introduction

The Ambient Air Quality Directives of European Union (EU) set standards for key air pollutants. These values take into account the 2005 WHO guidelines and considerations of technical and economic feasibility at the time of their adoption.

Air quality forecasting, if reliable and sufficiently accurate, can play an important role as part of an air quality management system (NOAA, 2001). Its applications can fall into several broad areas, such as health alerts—many cities currently provide warnings to the public when air pollution levels exceed specified levels, being those warnings directed at specific populations that are particularly sensitive to air pollution (e.g., asthmatics) (Liu et al., 2018); in addition, air quality forecasts can supplement existing emission control programs or emergency responses, with cities offering free access to public transportation (Quarmby et al., 2019); on pollution episode days, to reduce vehicle emissions, and regions implementing the “No-Burn day” (AQMD, 2022); consisting of a ban period on wood-burning in residential fireplaces, stoves, or outdoor fire pits, when particulate matter concentrations are expected to reach unhealthy levels, due to air emissions and stagnant weather conditions.

To predict the next-day daily average concentrations of particulate matter (PM_10_ and PM_2.5_), daily hourly maximum concentrations of ozone (O_3_), and daily hourly maximum concentrations of nitrogen dioxide (NO_2_), at air quality monitoring stations locations, forecast models were developed based on statistical methods using multiple linear regression (MR) and Classification and Regression Tree (CART) analysis. The NOVA University Lisbon (NOVA School of Science and Technology), in collaboration with the Portuguese Environment Agency (APA) and the Portuguese Institute for Sea and Atmosphere (IPMA), runs and disseminates daily air quality forecasts based on a statistical approach, first used by Cassmassi (1987) at South Coast Air Quality Management District California, USA. This statistical methodology is now in operation, in Portugal (Neto et al., 2005), for more than a decade and is the subject of regular updates, reflecting the ongoing research, and the changes in the air quality monitoring network. Recently the same methodology was extended to Madeira Autonomous Region, in Portugal, and was also applied to Macao Special Administrative Region of the People's Republic of China (MSAR), resulting from a collaboration with the Macao Meteorological and Geophysical Bureau (SMG) (Lei et al., 2019, 2020).

Air pollution is a major concern issue for most countries in the world. The global burden of disease associated with air pollution exposure exacts massive toll on human health worldwide: it is estimated to cause millions of deaths and lost years of healthy life annually. The burden of disease attributable to air pollution is now estimated to be on a par with other major global health risks, such as unhealthy diet and tobacco smoking, and air pollution is now recognized as the single biggest environmental threat to human health (World Health Organization, 2021). In Portugal, despite improvements in the past two decades, there are still exceedances mainly to nitrogen dioxide (NO_2_) annual limit value, to particulate matter (PM_10_) daily limit value, and to ozone (O_3_) target value. In Macao, concentrations of these pollutants are frequently above the thresholds accepted as “good air quality.”

High concentrations of NO_2_, PM, and O_3_ in the low troposphere are an additional risk factor for cardiovascular and respiratory diseases and contribute to mortality all over the world (Sheng and Tang, 2013; Lee et al., 2017). Surface O_3_ is known by its negative impacts in the respiratory system leading to more hospitalizations (Entwistle et al., 2019). PM, in particular smaller fractions such as PM_2.5_, are a major concern once they can get deep into lungs and some may even get into the bloodstream (Wiśniewska et al., 2019). Finally, specific combinations of concentration levels of these pollutants may be more dangerous than equally high levels of all the pollutants (Sheng and Tang, 2013). Portugal follows the European Union (EU) legislation (Directive 2008/50/EC) (European Union Legislation, 2008) on air quality, and Macao follows the Chinese National Ambient Air Quality Standards (NAAQS), which in turn are based in the Interim target-1 Air Quality Guidelines from the WHO (WHO, 2006). EU limit values, legally binding, were set for human health protection for, among other pollutants and averaging periods, PM_10_ as 50 μg/m^3^ (at a daily basis), PM_2.5_ as 25 μg/m^3^ (at annual basis), NO_2_ as 40 μg/m^3^ (at annual basis), and the O_3_ target value is of 120 μg/m^3^ (referring to the maximum daily 8-h mean, represented as O_3MAX_). The NAAQS has set the threshold of PM_10_ at 150 μg/m^3^ (daily basis), PM_2.5_ at 75 μg/m^3^ (daily basis), NO_2_ at 40 μg/m^3^ (annual basis), and O_3MAX_ at 160 μg/m^3^ (daily maximum 8-h mean). Some of these values contrast with the recently recommended WHO Air Quality Guideline levels, updated in 2021, after a systematic review of accumulated evidence, which are set at 45 μg/m^3^ for PM_10_ (daily basis), 15 μg/m^3^ (daily basis) and 5 μg/m^3^ (annual basis) for PM_2.5_, 10 μg/m^3^ for NO_2_ (annual basis), and 100 μg/m^3^ for O_3_ (daily maximum 8-h mean) (World Health Organization, 2021).

High-density and high-rise cities have become increasingly common in Asia (Lee et al., 2017). Air quality is a significant public health risk in many of these cities (World Health Organization, 2016). Macao, a coastal city located in southern China, is one example of a high-density, high-rise city with air quality issues. Macao was listed as the number one most densely populated region in the world (Sheng and Tang, 2013), with a population density of about 20,000 inhabitants/km^2^, accounting for a population of 680,000 within an area of 32.9 km^2^. The clustering effect is further enhanced by the prevalence of high-rise buildings. European cities likely have lower pollution and building densities, and fewer small-scale dispersed pollution sources, than high-density high-rise cities.

Factors leading to variation in pollution levels are diverse, and include both human activities and meteorological factors (Boubel et al., 1994). Meteorology plays a fundamental role in the re-distribution of air pollutants after their release in the atmosphere (Boubel et al., 1994). The characterization of local and large scale circulation winds and vertical atmospheric stability, allows accounting for transport, mixture, and dispersion processes (Boubel et al., 1994). Precipitation refers to the natural processes by which material is removed by atmospheric hydrometeors (cloud and fog drops, rain, and snow) and delivered to the Earth's surface (Seinfeld and Pandis, 2006).

The association between specific meteorological parameters and air quality can be quantified using a variety of statistical techniques. Statistical forecast methods analyze the events without knowing the mechanism of the change; therefore, this method is not dependent on physical, chemical, or biological processes (Bai et al., 2018). Instead, methods, such as regression analysis, investigate relationships between variables. Forecasting is a requisite part in the science of big data, and can be used to infer the future development of an object relative to previous information (Bai et al., 2018). Pollution forecasting can be understood as an estimation of a pollutant concentration at a specified future date.

This work aimes to provide an overall description of the current statistical methods, used by NOVA University Lisbon air quality group, to forecast air pollutant concentrations. Some of the discussed aspects are related with data requirements, steps involved in the model development, advantages and disadvantages of this approach. Model performance indicators are presented for each region and pollutant. Finally, examples of model performance are presented, for pollution episodes occurred in 2019 over the three studied regions. In this context, air quality forecast models are relevant tools because of their ability to anticipate and follow pollution episodes, allowing to support decisions, such as early warnings to the population, which can take preventive measures and avoid exposure, and reducing negative health impacts.

## Methods

Nowadays, forecasting, by statistical methods, has a wide range of applications and is used all over the world, based on the application of a multitude of algorithms. These methods are very accurate and enable a better understanding of the relationships between air quality data behavior and the underlying meteorology (Bai et al., 2018). In the present work, statistical models were developed based on the techniques of MR and CART. Both techniques rely on the historical data of meteorological and air quality variables.

Regression analysis methods are based on the association between pollutant levels, and meteorological and aerometric variables, which can be quantified by analyzing historical datasets, using standard statistical analysis packages, as shown on previous works (Cassmassi, 1987; US EPA, 2003; Choi et al., 2013; Durão et al., 2016; Oduro et al., 2016). The resultant multivariant linear regression equation is then used to forecast future pollution levels. The CART technique identifies those variables (meteorological or air quality) that are most strongly correlated with ambient pollution levels. These variables are then used to predict next day pollution levels, either daily average or maximum hourly concentrations depending on the pollutant, based on same day air quality levels and next day forecasted meteorology.

**Figure 1:**
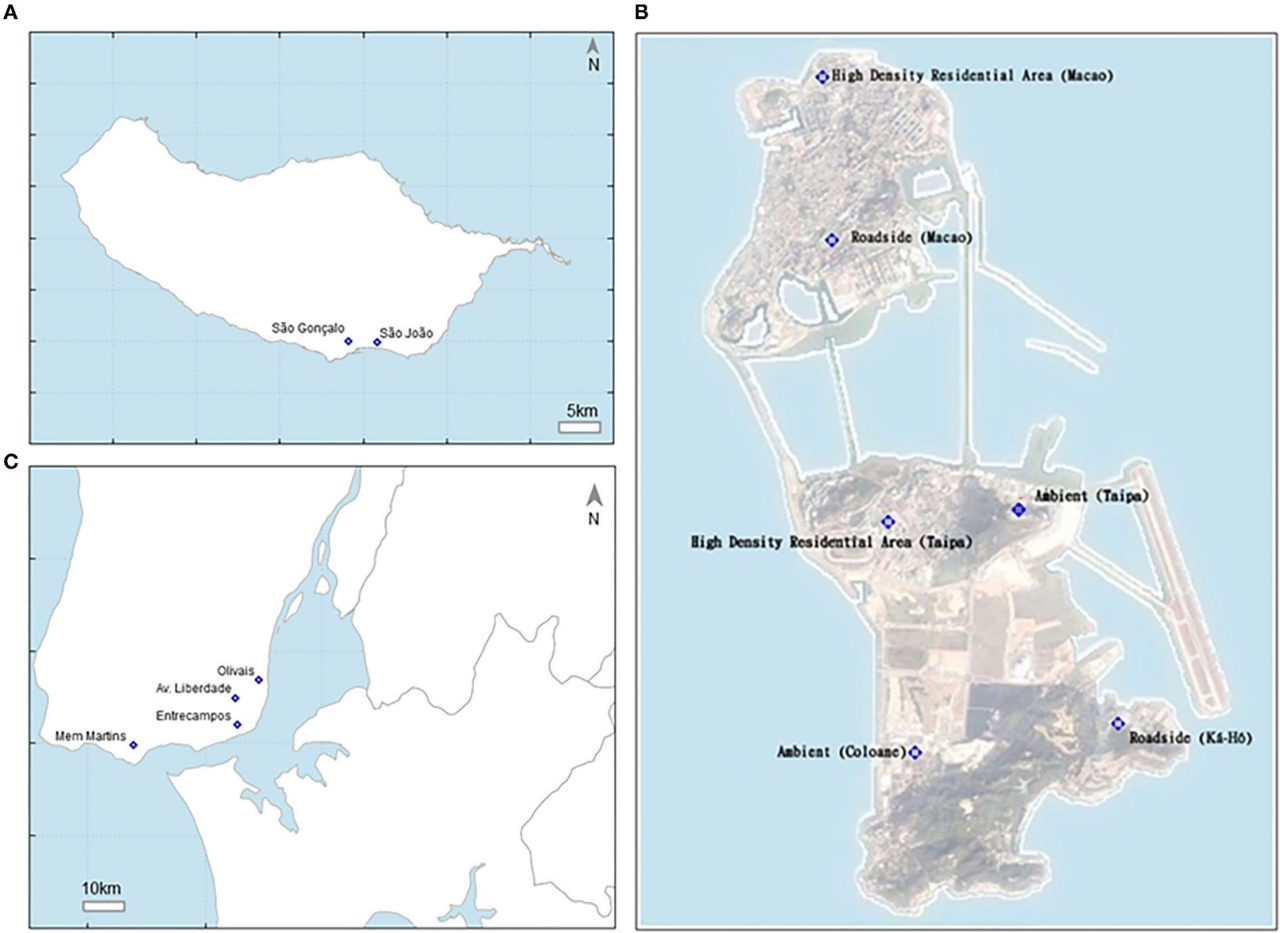
**(A)** Madeira island air quality observation network (modeled subset); **(B)** Macao air quality observation network (source: https://www.smg.gov.mo/en/subpage/182/page/123); and **(C)** Lisbon air quality observation network.

The referred statistical models were applied to forecast the average daily concentrations of PM_10_ and PM_2.5_, and the hourly maximum concentrations of O_3_ and NO_2_ (referred as O_3MAX_ and NO_2MAX_, respectively), for the next day, for each air quality monitoring station (AQMS) location. For the work presented here, a set of AQMS were selected in the Greater Lisbon Area (4 AQMS), Madeira Autonomous Region (2 AQMS), and Macao (4 AQMS), represented in Figure 1.

The Greater Lisbon Area includes the Portuguese capital, Lisbon, being the main economical sub region of the country. It covers 1,376 km^2^ and it is the most densely populated Portuguese sub region (with 2,042,477 inhabitants and 1,484 inhabitants/km^2^). The Greater Lisbon Area presents a mild subtropical or a hot-summer Mediterranean climate (Csa), according to Köppen's classification (Köppen, 1936), based on temperature and precipitation mean values. Lisbon summer is mild to hot with significant temperature variations related, in part, to coastal distance. The wind blows most frequently from the north quadrant, according to the 1971–2000 climatological normals (IPMA, 2022).

Madeira, an autonomous region of Portugal, is an archipelago comprising four islands off the northwest coast of Africa. This region comprises a population of 253,945 inhabitants within an area of 801 km^2^. The capital of Madeira is Funchal that has a subtropical Mediterranean climate (Csa), according to Koppen's classification (Köppen, 1936). Funchal's climate is predominantly determined by the Atlantic Ocean and as a result weather extremes are rare. Temperature usually rises significantly when the influence of the persistent eastern-winds from northern Africa is felt. The annual average maximum temperature is 22.1°C and the average of minimum temperature is 15.8°C (IPMA, 2022).

Located along the southeast coast of Mainland China, Macao is surrounded by water on three sides, with a subtropical oceanic monsoon climate that is characterized by high temperatures, high levels of atmospheric moisture and abundant rainfall (SMG, 2022). In winter, Macao is cold and dry with predominant northern winds, and the summer is presented with heavy rains due to the strong southwest monsoon. Spring and autumn are transition periods. The winter northeast monsoon is known to have the ability to transport pollutants from northern and eastern China (Tong et al., 2018). In summer season, from June to August, rainfall increases, providing a better atmospheric mixing, and persistent southern winds occur, resulting in PM levels to decrease (Lopes et al., 2016).

Data from 3- to 6-year daily series observations, were used to develop the forecast models, and each of the models was evaluated using 2019 data. The time period used to build each model equation is different for each region and AQMS, according to data availability (Table 1). The selection of a representative modeling period is important, being recommended at least 2 years of data.

**Table 1:** Modeling and validation periods considered by region and air pollutants forecasted at each air quality monitoring station (AQMS).

**Region**	**Modeling** **period**	**Validation** **period**	**AQMS** **(Type)**	**Modeled** **Pollutants**
Greater Lisbon Area	2015–2018	2019	Av. Liberdade (UT)	PM_10_, NO_2_
			Entrecampos (UT)	PM_10_, PM_2.5_, NO_2_
			Olivais (UB)	PM_10_, PM_2.5_, NO_2_, O_3_
			Mem Martins (UB)	PM_10_, PM_2.5_, NO_2_, O_3_
Madeira autonomous region	2016–2018	2019	São João (UT)	PM_10_, PM_2.5_, NO_2_
			São Gonçalo (UB)	PM_10_, O_3_
Macao administrative region	2013–2018	2019	Macao Roadside (UT)	PM_10_, PM_2.5_, NO_2_
			Macao Residential (HDR)	PM_10_, PM_2.5_, NO_2_, O_3_
			Taipa Ambient (UB)	PM_10_, PM_2.5_, NO_2_, O_3_
			Taipa Residential (HDR)	PM_10_, PM_2.5_, NO_2_, O_3_

*AQMS, Air Quality Monitoring Station; UT, Urban Traffic; UB, Urban Background; HDR, High Density Residential*.

**Figure 2:**
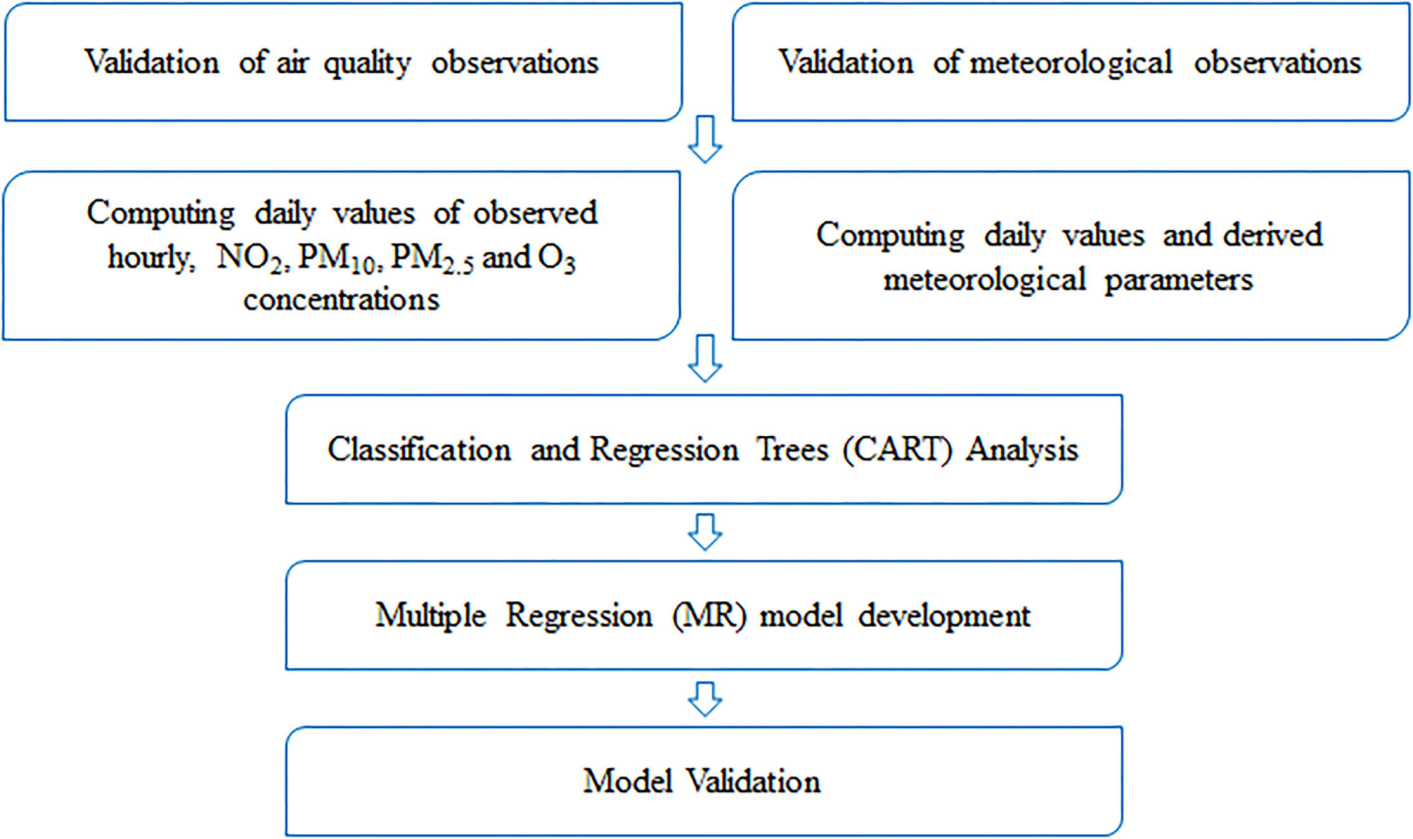
Flowchart for the model development of air quality forecast by statistical methods.

Regarding the data collection phase, a large set of meteorological and air quality data was gathered, namely: (i) meteorological surface observations: hourly observations from automatic weather stations, such as temperature, relative humidity, and dew point temperature; (ii) upper-air observations, such as, geopotential heights, temperature, relative humidity, and dew point temperature at various altitudes; and (iii) surface air quality measurements, from AQMS network, of PM_10_, PM_2.5_, NO_2_, and O_3_. Other variables were added to the analysis, as the flag for week/weekend day and the daily sunlight period duration. A list of independent variables and data sources used as potential predictors in the modeling phase is presented on Table 2. The model development flowchart is represented in Figure 2.

**Table 2:** Data sources and variables on a daily temporal scale used in the modeling process.

**Data type**	**Source**	**Variables**	**Description**
Meteorological data	Upper air meteorological observations (Aerological soundings)	H_1000, H_850, H_700, H_500	Geopotential height at pressure levels (indicator of synoptic-scale weather pattern) (hPa)
		TAIR_925, TAIR_850 and TAIR_700	Air temperature at pressure levels (measure of the strength and height of subsidence inversion) (°C)
		RH_925, RH_850, RH_700	Relative humidity at pressure levels (%)
		DEWP_925, DEWP_850, DEWP_700	Dew point at pressure levels (°C)
		THI_850, THI_700, THI_500	Thickness at pressure levels (associated to the mean temperature in the layer)
		STB_925, STB_850, STB_700	Stability at pressure levels (detector of atmospheric stability)
	Surface meteorological observations (hourly data)	TAIR_MEA_, TAIR_MIN_, TAIR_MAX_	Air temperature, mean, minimum and maximum (air stability and emission rates from engines) (°C)
		RH_MEA_, RH_MAX_, RH_MIN_	Relative humidity, daily mean, maximum and minimum values (%)
		DEWP_MEA_	Mean dew point (°C)
		V_MEA_, V_MAX_	Wind speed mean and maximum values (horizontal dispersion) (m/s)
		PREC	24 h Accumulated Precipitation (pollutant removal indicator) (mm)
		STA1_P-STA2_P	Pressure difference between stations (indicator of synoptic scale weather) (hPa)
Air quality data	Surface air quality stations (hourly data)	PM_10__D1, PM_10__D2, PM_10__D3, PM_10__D12, PM_2.5__D1, PM_2.5__D2, PM_2.5__D3, PM_2.5__D12	Daily mean concentrations for particulate matter (PM_10_ and PM_2.5_) for the recent past (last 3 days—D1 to D3) and the last 24h from each days noon (D12) (μg/m^3^)
		O_3__D1, O_3__D2, O_3__D3, O_3__D12, NO_2__D1, NO_2__D2, NO_2__D3, NO_2__D12	Daily maximum concentrations for ozone (O_3_) and nitrogen dioxide (NO_2_) for the recent past (last 3 days—D1 to D3) and the last 24h from each days noon (D12) (μg/m^3^)
Other data	Geographical data and human behavior descriptors	Daylight	Number of hours of daylight (h)
		WW	Week/Weekend indicator flag (human activity and traffic)

Series with a low annual efficiency (<75%) on data availability were rejected. For operational purposes, limitations related to the expected daily data availability, to perform the forecast, were also considered. Outliers were identified and excluded from the data series.

In the process of the MR model development, several of the initial variables were removed of the final models due to high correlation values among variables, or due to the commitment to obtain the simplest model, with the smallest number of variables that maximizes the explained variance. In addition, fewer variables mean that, in operational mode, missing data have a lower impact on the quality of the forecast. In MR analysis, one has to seek for a compromise between model improvement, obtained by adding variables, and the increase of complexity and uncertainty introduced by a new variable.

Another important aspect is the effort to achieve accurate forecast results when higher concentrations are predicted, since they are related with higher negative health impacts. One of the advantages of the CART technique is to be able to establish particular model equations that can accommodate specific trends caused by meteorological circumstances that trigger high level concentrations (Choi et al., 2013). This is important to send advisory recommendations in the anticipation of higher pollution episodes to avoid excessive exposure.

**Figure 3:**
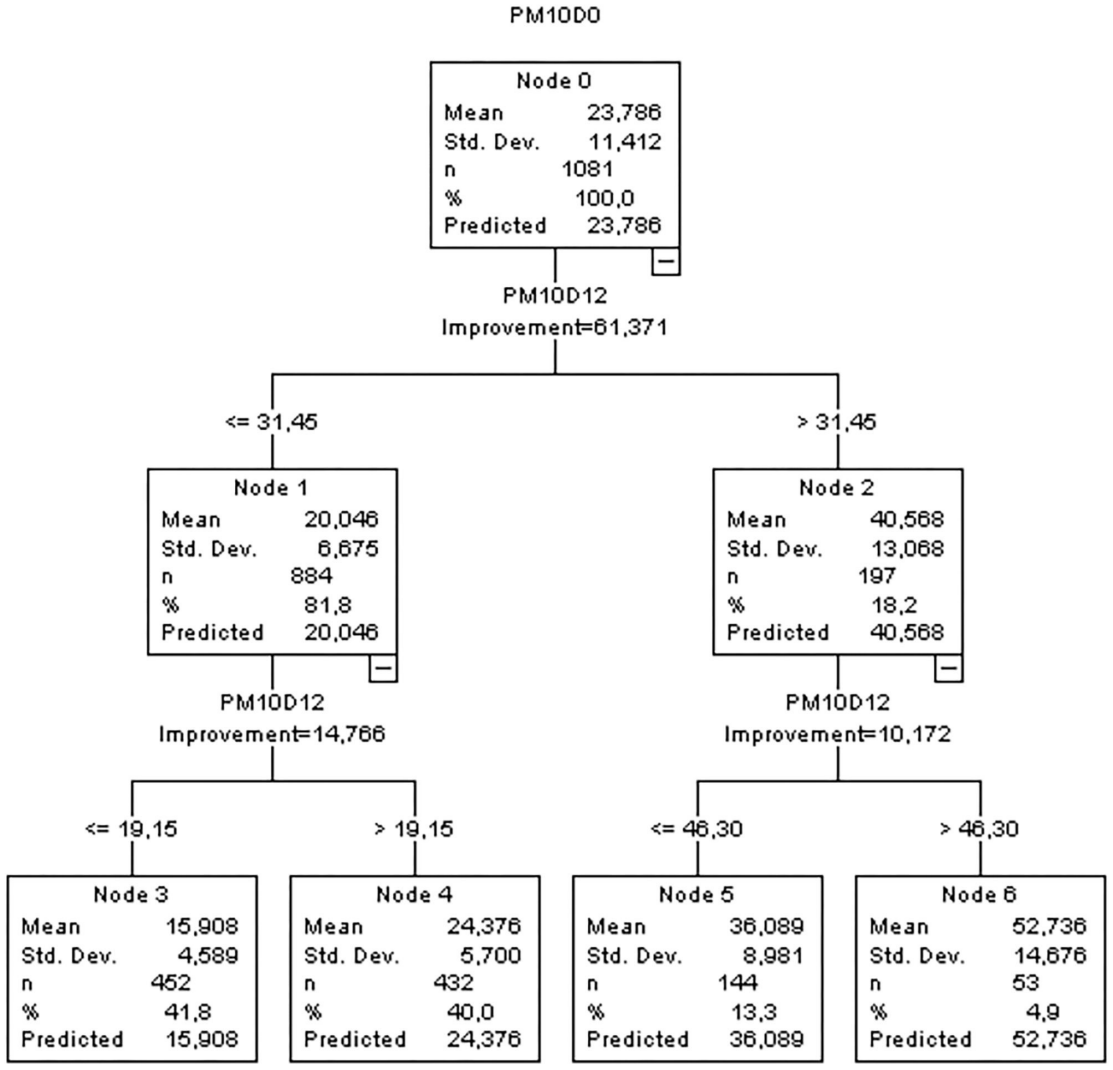
Classification and Regression Tree (CART) analysis obtained for PM_10_, Entrecampos.

The CART analysis defines a path with several nodes, where threshold values on specific variables split into binary ramifications, based on the largest reduction in variations in the target variable in each of the new branch (Choi et al., 2013). CART analysis produces a tree representation, as exemplified in Figure 3 for PM_10_, according to some parameterizations, as the pretended tree depth. The CART analysis and the MR model development were performed using the IBM software SPSS (Version 25).

Model performance evaluation was accomplished by computing the most common scores: (i) bias (Equation 1), (ii) mean absolute error (MAE) (Equation 2), (iii) root mean square error (RMSE) (Equation 3), (iv) coefficient of determination (*R*^2^) (Equation 4), and (v) relative mean absolute error (RMAE) (Equation 5), where *f* represents the forecasting value, *o* the observed value, *n* the forecast/observation pairs, *f* the forecasting mean value, and *o* the observed mean value. These statistic measures of agreement were obtained by comparing the forecasted 2019 validation data set to the observed/monitored air pollutant levels on that year.


(1) $$\begin{array}{r} BIAS\;=\;\frac{1}{n}\overset{n}{\underset{i=1}{\sum }}\left(f_{i}-o_{i}\right) \end{array}$$

(2) $$\begin{array}{r} MAE\;=\;\frac{1}{n}\overset{n}{\underset{i=1}{\sum }}|f_{i}-o_{i}| \end{array}$$

(3) $$\begin{array}{r} RMSE\;=\;\sqrt{\frac{1}{n}}\overset{n}{\underset{i=1}{\sum }}{\left(f_{i}-o_{i}\right)}^{2} \end{array}$$

(4) $$R^{2}=\frac{[\int_{i=1}^{n}(f_{i}–\bar{f})–{(o_{i}–\bar{o})]}^{\wedge }2}{2[\int_{i=1}^{n}{(f_{i}–\overset{¯}{f})}^{\wedge }2]\text{ [}\int_{i=1}^{n}{(o_{i}–\bar{o})}^{\wedge }2]}$$

(5) $$\begin{array}{r} R_{MAE}\;=\;{\left(\frac{MAE}{\;\bar{o}}\right)}^{*}100 \end{array}$$

The obtained statistical models allow to perform a daily forecast, for the next day, of PM_10_, PM_2.5_, NO_2_, and O_3_ concentrations, in an operational mode, for the three studied regions. The prediction models run daily, after 16 h for Macao and 13 h for both Greater Lisbon Area and Madeira Autonomous Region, according to the daily schedules in which air quality data is made available.

In the final stage of the operational process, a forecasted air quality index (AQI) is produced for each pollutant for the next day based on the daily air pollutant concentrations, mean or maximum daily values, depending on the pollutant. The final AQI, for each location, corresponds to the worst level of air quality among the forecasted pollutants.

## Results

### Model Selected Variables and Performance Indicators

The statistical models based on MR and CART analysis were developed to forecast NO_2_, PM_10_, PM_2.5_, and O_3_ concentrations. The objective is to perform a daily forecast, for the next day, in an operational mode by running the prediction models after 16 h for Macao and 13 h both Greater Lisbon Area and Madeira Autonomous Region.

CART analysis was tested mainly to better predict high concentration levels. For Macao region, CART analysis did not improve the quality of overall predictions being, in this case, the prediction models based only on one MR equation. We believe that in Macao, pollution is frequently due to distant sources with pollutants being, transported through the advection of air masses by large scale circulation. Therefore, local meteorology is not as critical, being one equation sufficient to explain and enable the prediction of next day pollutant concentrations, for each monitoring location. The exception was verified for O_3MAX_ predictions, at two AQMS. In these last cases, CART analysis allowed to identify split nodes, for which O_3_ prediction equations were determined, afterward, by using MR for each node. Opposing to Macao trend, in Greater Lisbon Area, almost every AQMS and pollutant are being forecasted with CART and MR.

The most prevalent variable, being selected at all the forecast equations, is the one that represents the last 24-h pollutant concentrations (16 h from yesterday to 15 h today in the case of Macao, and 11 h of yesterday to 12 h of today for the Great Lisbon Area and Madeira Autonomous Region). Regarding the meteorological selected independent variables used as predictors (Table 3), the geopotential height at 850 hPa (H_850), indicator of synoptic-scale weather pattern, is frequently present in the forecast of NO_2_ and PM, both in Lisbon and Macao. For Lisbon and Madeira stations, the most common and frequent weather variable is air temperature at 925 hPa (TAIR_925), a measure of the strength and height of the subsidence inversion, especially for PM forecasts. In Macao, in addition to H_850 (the most common variable), figures the RH_MEA_, attributing relevance to relative humidity in the air quality forecast at this region. In Lisbon, the final set of model selected variables covers 13 weather variables. In Madeira and Macao, there is a lower variability of different weather variables: for Madeira TAIR_925 and the average dew point temperature (DEWP_MEA_) are the most common with more four different variables, and in Macao H_850 and RH_MEA_ are the most common with more seven different variables. In Table 4, the obtained MR model equations are presented for one AQMS selected for each studied region.

**Table 3:** Forecast model selected variables for each pollutant and air quality monitoring station.

**Location/AQMS/pollutant**			**Model selected weather variables**																
			**H**			**TAIR 925**	**RH 925**	**DEWP 700**	**THI 700**	**STB**			**TAIR**		**RH**		**DEWP MEA**	**V-MAX**	**PREC**
			**1,000**	**850**	**500**					**925**	**850**	**700**	**MAX**	**MIN**	**MEA**	**MIN**			
Greater Lisbon Area	ENT	PM_10_		x			x												x
	AVL			x		x												x	
	OLI			x		x								x					
	MEM			x	x	x													
	ENT	PM_2.5_		x															
	OLI					x													x
	MEM			x		x	x							x					
	ENT	NO_2_		x													x	x	
	AVL			x	x		x					x				x			
	OLI		x	x												x			
	MEM		x	x														x	x
	ENT	O_3_							x			x	x					x	
	OLI				x				x			x	x	x					
	MEM							x				x						x	
Madeira Administrative Region	SJO	PM_10_				x											x		
	SGO					x											x		
	SJO	PM_2.5_				x	x												
	SJO	NO_2_			x	x											x	x	
	SGO	O_3_			x											x			
Macao Autonomous Region	M_RES	PM_10_		x											x				
	M_ROA			x											x				
	T_AMB			x											x				
	T_RES			x											x				
	M_RES	PM_2.5_		x													x		
	M_ROA			x											x				
	T_AMB			x											x				
	T_RES					x											x		
	T_RES	NO_2_		x												x			
	M_ROA			x												x			
	T_AMB			x						x									
	T_RES			x													x		
	M_RES	O_3_					x						x						
	T_AMB										x								
	T_RES			x												x			

*ENT, Entrecampos; AVL, Avenida da Liberdade; OLI, Olivais; MEM, Mem Martins; SJO, São João; SGO, São Gonçalo; M_RES, Macao Residential; M_ROA, Macao Roadside; T_AMB, Taipa Ambient; T_RES, Taipa Residential; H, Geopotential height at different pressure levels; TAIR, Air temperature 925 hPa; RH, Relative humidity at 925 hPa; DEWP, Dew Point at 700 hPa; THI, Thickness at 700 hPa, STB, Stability at different pressure levels; TAIR, Temperature, maximum and minimum; RH, Relative humidity, mean and minimum; DEWP_MEA_, Mean dew point; V_MAX, Maximum wind speed; PREC, Precipitation*.

**Table 4:** Model equations obtained for Greater Lisbon Area (Entrecampos), Madeira Autonomous Region (São João), and Macao Administrative Region (Taipa Ambient).

**Station**	**Pollutant**	**Model equations**
Greater Lisbon Area: Entrecampos	NO_2MAX_	**CART nodes and multiple regression equations:**
		**For NO**_**2**_**D12** **≤** **75.45**
		NO_2MAX_ = NO_2_D12 * 0.784 – (STA1_P – STA2_P) * 5.201 + H_850 * 0.015
		**For 75.45** **<** **NO**_**2**_**D12** **≤** **120.05**
		NO_2MAX_ = NO_2_D12 * 0.424 + H_850 * 0.048 – V_MAX_ * 2.169
		**For NO**_**2**_**D12** **>** **120.05**
		NO_2MAX_ = NO_2_D12 * 0.311 + H_850 * 0.068 – ${\text{TAIR}}_{\text{MEA}}^{*}$ 2.303
	PM_10_	**CART nodes and multiple regression equations:**
		**For PM**_**10**_**D12** **≤** **31.45**
		PM_10_ = PM_10_D12 * 0.787 + H_850 * 0.003 – (STA1_P – STA2_P) * 0.717 + TAIR_925 * 0.122
		**For 31.45** **<** **PM**_**10**_**D12** **≤** **46.30**
		PM_10_ = PM_10_D12 * 0.965 – PREC * 0.421
		**For PM**_**10**_**D12** **>** **46.30**
		PM_10_ = H_850 * 0.041 – RH_925 * 0.239
	PM_2.5_	**CART nodes and multiple regression equations:**
		**For PM**_**2.5**_**D12** **≤** **18.85**
		PM_2.5_ = PM_2.5_D12 *0.860 + H_850*0.001
		**For 18.85** **<** **PM**_**2.5**_**D12** **≤** **27.25**
		PM_2.5_ = PM_2.5_D12 * 0.975
		**For PM**_**2.5**_**D12** **>** **27.25**
		PM_2.5_ = PM_2.5_D12 * 0.873
	O_3MAX_	**For O**_**3**_**D12** **≤** **51.20**
		O_3MAX_ = V_MAX_ * 2.122 – STB_700 * 1.511
		**For 51.20** **<** **O**_**3**_**D12** **≤** **75.55**
		O_3MAX_ = O_MAX_D12 * 0.707 + TAIR_MAX_ * 2.144 + THI_700 * 1.595
		**For O**_**3**_**D12** **>** **75.55**
		O_3MAX_ = O_3_D12 * 0.490 – STB_700 * 1.945 + WW * 9.065
Madeira Autonomous Region: São João	NO_2MAX_	**Multiple regression equation:** NO_2MAX_= NO_2_D12 * 0.7950 + H_500* (0.0030) – DEWP_MEA_ * 0.6160 – WW * 1.4930 – Daylight * 0.4520 + TAIR_925 * 0.4380 – V_MAX_ * 0.1950 – STB_850 * 0.1370
	PM_10_	**Multiple regression equation:** PM_10_ = PM_10_D12 * 0.9360 + TAIR_925 * (0.4130) – PM10D12 * 0.0960 - DEWP_MEA_ * 0.2520 + PM_10_D3 * 0.0650 – WW * 0.9360
	PM_2.5_	**CART nodes and multiple regression equations:**
		**For PM**_**2.5**_**D12** **≤** **5.95**
		PM_2.5_ = PM_2.5_D12 * 0.895 + TAIR_925 * 0.060
		**For 5.95** **<** **PM**_**2.5**_**D12** **≤** **9.55**
		PM_2.5_ = PM_2.5_D12 * 1.079 – WW * 0.407 – RH_925 * 0.006
		**For PM**_**2.5**_**D12** **>** **5.95**
		PM_2.5_ = PM_2.5_D12 * 0.814 – WW * 1.703 + TAIR_MAX_ * 0.122
Macao Administrative Region: Taipa Ambient	NO_2_	**Multiple regression equation:** NO_2_ = NO_2__D16 * 0.914 + H_850 * 0.004 + STB-925 * 0.734
	PM_10_	**Multiple regression equation:** PM_10_ = PM_10__16D1* 0.905 + H_850 * 0.014 – RH_MEA_ * 0.205
	PM_2.5_	**Multiple regression equation:** PM_2.5_ = PM_2.5__16D1* 0.928 + H_850 * 0.006 – RH_MEA_ * 0.093
	O_3MAX_	**CART nodes and multiple regression equations:**
		**For O**_**3**__**MAX**_**_16D1** **≤** **105.50**
		O_3MAX_ = O_3MAX__16D1 * 1.034 – O3_MAX__23D1 * 0.214 + H_850*0.019 – RH_MIN_ * 0.236
		**For 105.50** **<** **O**_**3MAX**_**_16D1** **≤** **181.87**
		O_3MAX_ = O_3MAX__16D1 * 0.994 – O_3MAX__23D1 * 0.433 + H_850 * 0.051 – RH_MIN_ * 0.529
		**For O**_**3MAX**_**_16D1** **>** **181.87**
		O_3MAX_ = O_3MAX__16D1 * 1.006 – O_3MAX__23D1 * 0.473 – STB_850 * 8.608

**Figure 4:**
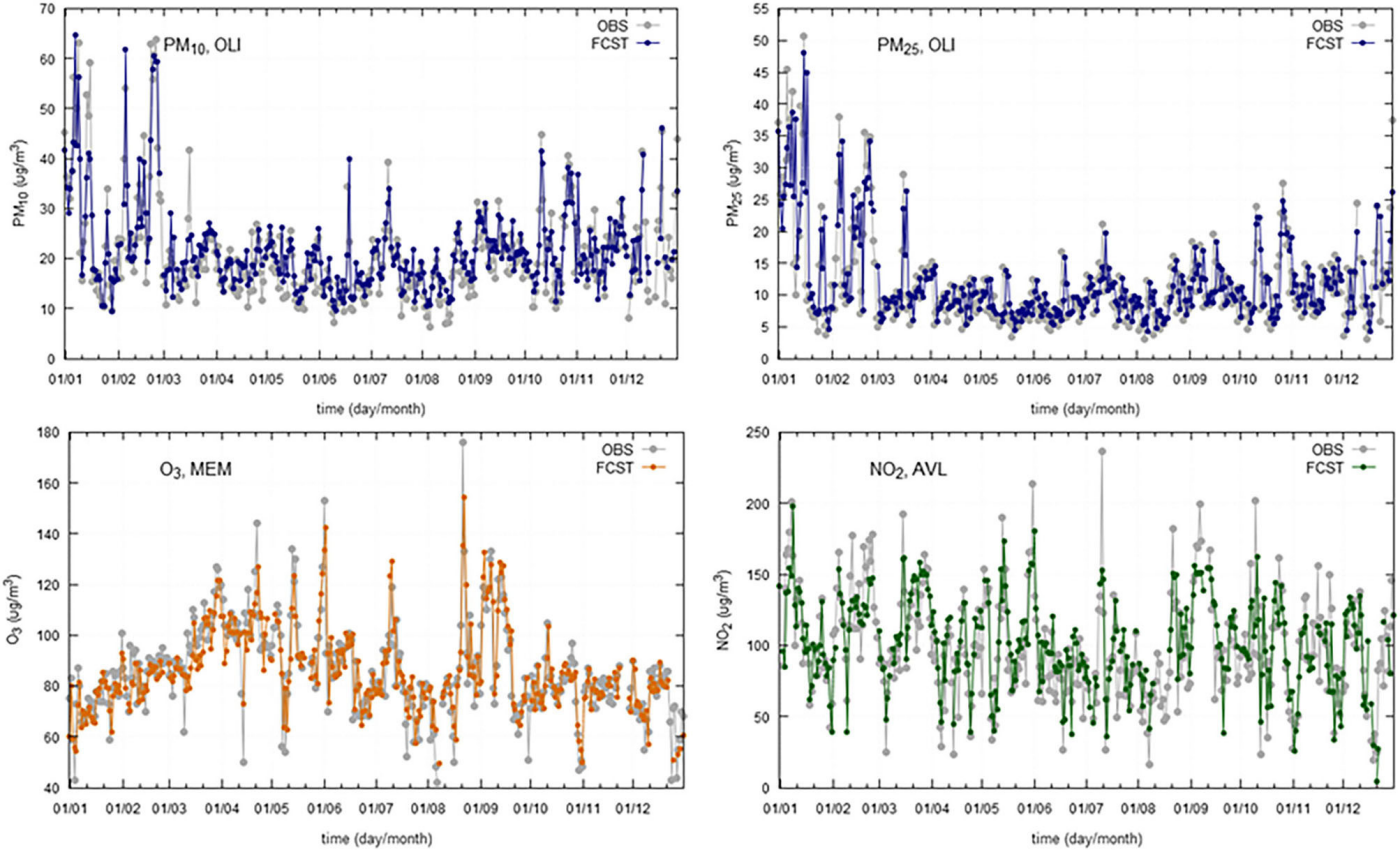
Daily observations (OBS) and forecasts (FCST) at three monitoring stations (AVL, Avenida da Liberdade; MEM, Mem Martins; ENT, Entrecampos) in Greater Lisbon Area, for 2019.

**Figure 5:**
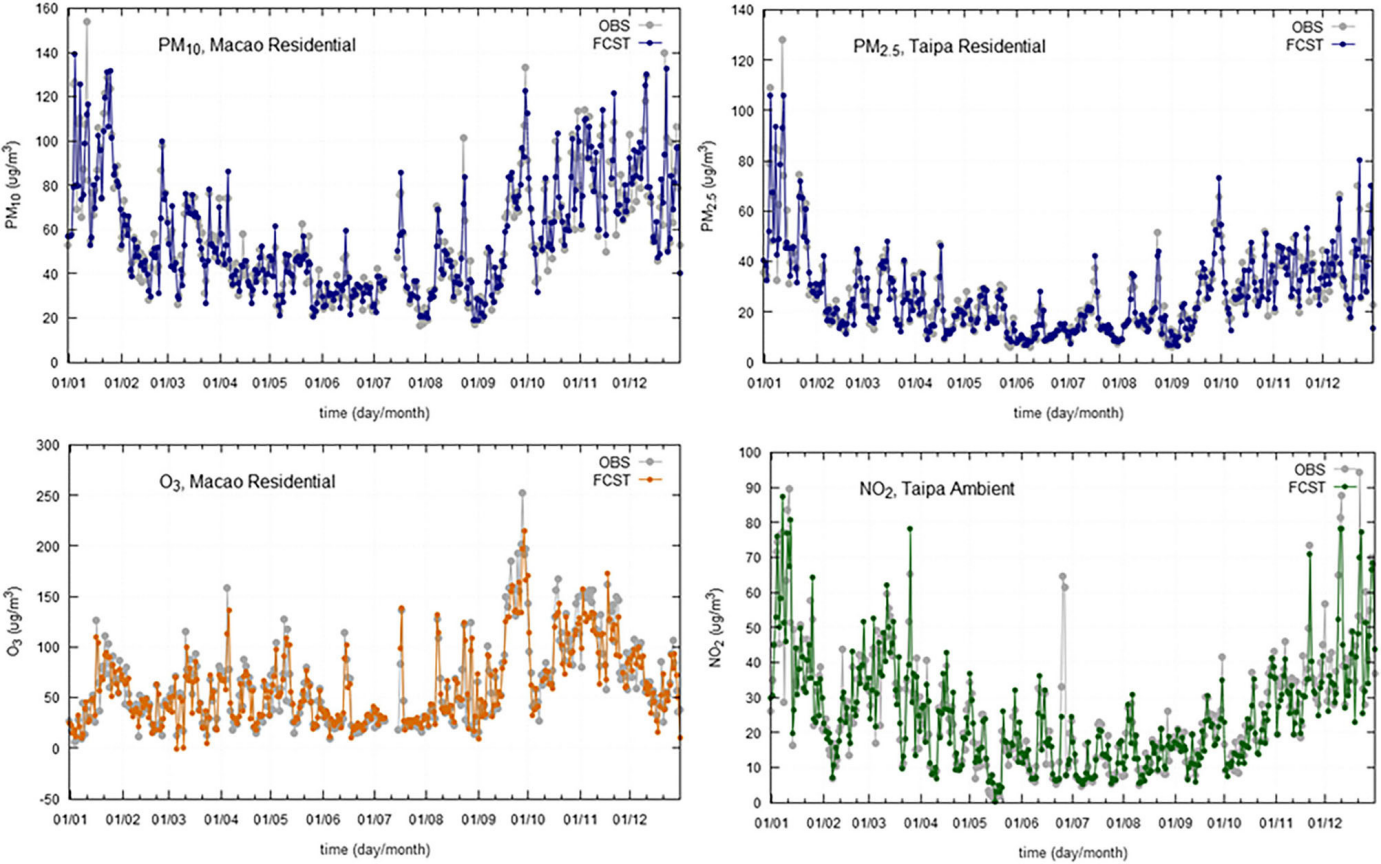
Daily observations (OBS) and forecasts (FCST) at three monitoring stations (Macao Residential, Taipa Residential, and Taipa Ambient) in Macao Administrative Region, for 2019.

**Figure 6:**
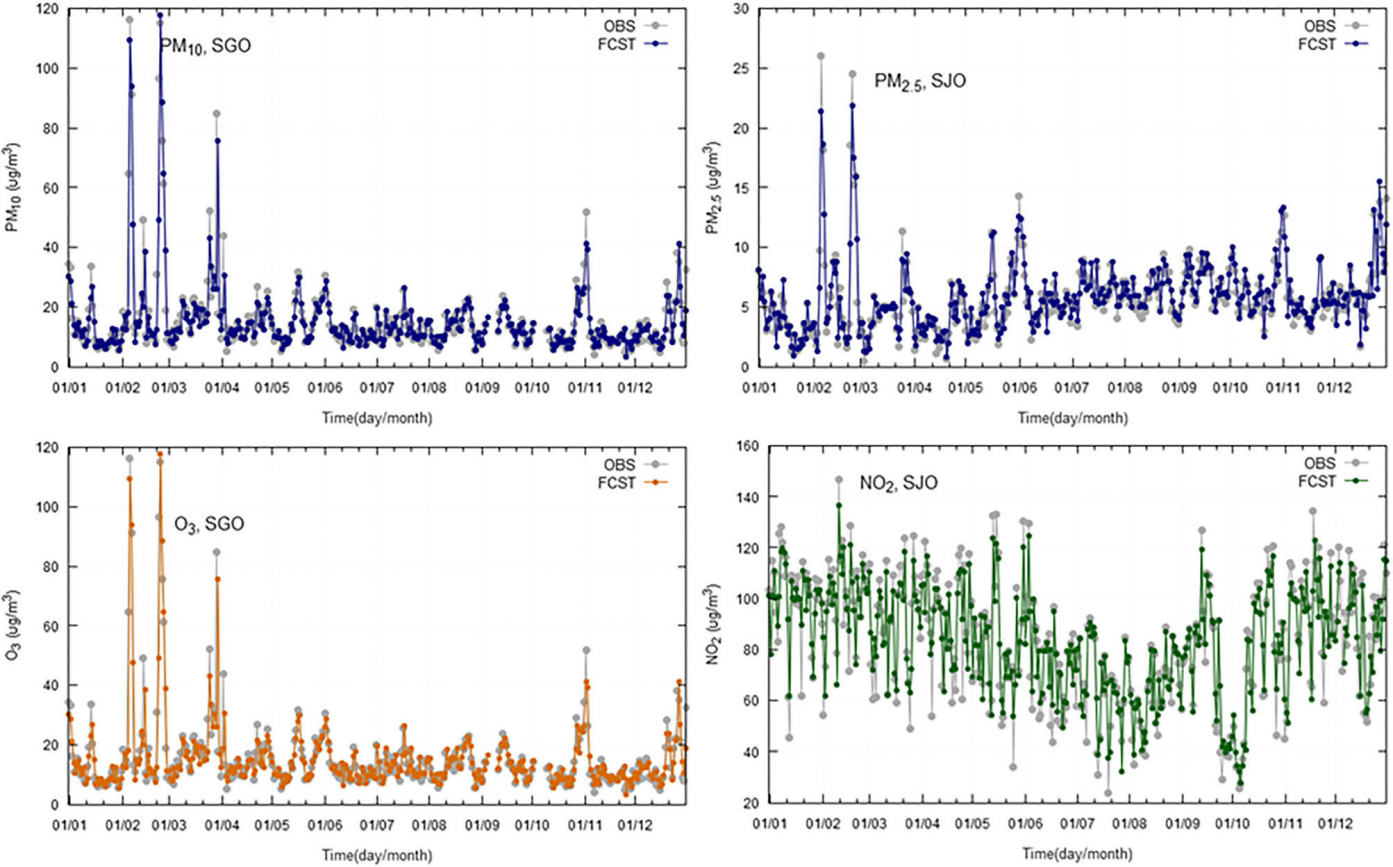
Daily observations (OBS) and forecasts (FCST) at two monitoring stations (SJO, São João; SGO, São Gonçalo) in Madeira Autonomous Region, for 2019.

Models were validated with collected data from 2019. For validation purposes, it is important to use at least 1 year of data, to accommodate for all the seasonal variations. Model performance indicators are summarized, by region, in Table 5 (Greater Lisbon Area), Table 6 (Madeira Autonomous Region) and Table 7 (Macao Administrative Region). The referred tables contain the obtained model performance indicators, such as, *R*^2^, RMSE, MAE, Bias, and RMAE. For each station and pollutant, the forecasted time series was plotted against observations (Figures 4–6).

**Table 5:** Model performance indicators for validation with 2019 data, by AQMS and pollutant, at Greater Lisbon Area.

**Station**	**Type**	**Pollutant**	**Model performance indicators**					**Model type**
			***R* ^2^**	**RMSE**	**MAE**	**BIAS**	**RMAE (%)**	
Avenida da Liberdade	Urban traffic	PM_10_	0.79	5.1	3.8	1.1	14.6	CART + MR
		NO_2MAX_	0.62	23.7	18.3	2.6	17.9	CART + MR
Entrecampos	Urban traffic	PM_10_	0.76	5.0	3.6	0.9	16.5	CART + MR
		PM_2.5_	0.50	5.4	3.7	0.4	30.2	CART + MR
		NO_2MAX_	0.71	17.6	13.1	1.0	18.5	CART + MR
		O_3MAX_	0.62	11.6	8.8	2.6	12.8	CART + MR
Olivais	Urban background	PM_10_	0.71	5.3	4.1	1.6	21.3	CART + MR
		PM_2.5_	0.52	4.9	3.5	0.9	34.3	CART + MR
		NO_2MAX_	0.69	20.4	13.2	3.6	20.6	MR
		O_3MAX_	0.64	12.0	8.8	0.6	11.4	MR
Mem Martins	Urban background	PM_10_	0.81	3.1	2.3	0.1	12.9	CART + MR
		PM_2.5_	0.76	2.1	1.7	0.4	20.4	CART + MR
		NO_2MAX_	0.76	13.0	8.3	−0.2	27.4	MR
		O_3MAX_	0.66	10.8	8.0	−0.4	9.2	CART + MR

**Table 6:** Model performance indicators for validation with 2019 data, by AQMS and pollutant, at Madeira Autonomous Region.

**Station**	**Type**	**Pollutant**	**Model performance indicators**					**Model type**
			***R* ^2^**	**RMSE**	**MAE**	**BIAS**	**RMAE (%)**	
São João	Urban traffic	PM_10_	0.83	4.6	2.6	0.5	13.6	MR
		PM_2.5_	0.85	1.5	1.0	0.2	13.9	CART + MR
		NO_2MAX_	0.82	7.5	6.0	2.2	14.4	MR
São Gonçalo	Urban background	PM_10_	0.70	7.4	3.6	−0.2	23.6	CART + MR
		O_3MAX_	0.67	12.1	9.6	−3.2	9.5	MR

**Table 7:** Model performance indicators for validation with 2019 data, by AQMS and pollutant, at Macao Administrative Region.

**Station**	**Type**	**Pollutant**	**Model performance indicators**					**Model type**
			***R* ^2^**	**RMSE**	**MAE**	**BIAS**	**RMAE (%)**	
Macao Roadside	Urban traffic	PM_10_	0.88	8.4	5.6	1.5	11.8	MR
		PM_2.5_	0.87	5.2	3.3	0.2	13.6	MR
		NO_2_	0.89	7.9	5.8	−0.1	9.8	MR
Macao Residential	High density residential	PM_10_	0.89	8.8	5.9	−0.1	10.3	MR
		PM_2.5_	0.87	5.2	3.3	0.8	14.0	MR
		NO_2_	0.86	7.7	5.5	0.0	10.9	MR
		O_3MAX_	0.85	23.2	14.0	0.0	22.3	MR
Taipa Ambient	Urban background	PM_10_	0.88	7.8	5.1	0.8	14.3	MR
		PM_2.5_	0.86	4.8	3.1	0.2	17.7	MR
		NO_2_	0.87	6.1	4.2	1.0	16.3	MR
		O_3MAX_	0.86	23.7	14.7	−1.6	13.9	CART + MR
Taipa Residential	High density residential	PM_10_	0.88	7.9	5.1	0.2	8.7	MR
		PM_2.5_	0.88	5.6	3.5	−0.1	13.1	MR
		NO_2_	0.87	5.6	4.1	0.6	12.8	MR
		O_3MAX_	0.78	20.9	12.7	1.3	19.7	CART + MR

The results show a good agreement between modeled and observed concentrations, being statistically significant at the 95% *CI*. The selected models provide a good relationship between meteorological and air quality variables, when performing an air quality forecast under different situations.

The time series plotting displays a good overall correlation between observations and forecasted values, however, there is a slight trend to underestimate the maximum peaks. The statistical scores are comparable across the regions under analysis.

Regarding the obtained *R*^2^ for modeled vs. observed concentrations (Tables 5–7), the following aspects can be highlighted:

- the *R*^2^ is, on average, lower for Lisbon than for Madeira and Macao, ranging from 0.5 in Olivais for PM_2.5_ to 0.81 in Mem Martins for PM_10_;
- Macao presents *R*^2^-values ranging from 0.78 at Taipa Residential for O_3_ to 0.89 at Macao Roadside and Macao Residential (for NO_2_ and PM_10_, respectively);
- Madeira *R*^2^-values range from 0.67 at São Gonçalo for O_3_ to 0.85 at São João for PM_2.5_.

In general, the bias stays very close to zero with the maximum value being 3.6 achieved for the NO_2_ at the station Olivais (Lisbon). When comparing the BIAS and MAE, there are significant differences between pollutants, some of them related to different ranges of variation of the daily concentrations. The RMAE ranges from 8.7% for PM_10_ at Taipa Residential (Macao) to 34.3% for PM_2.5_, at Olivais. Comparing the RMAE for the different regions, Lisbon displays, on average, higher values than Macao and Madeira, being PM_2.5_ the pollutant with the lowest performance.

### Atmospheric Pollution Episodes

As examples of the response of developed models, in situations where air pollutant concentrations rise significantly, a few pollution episodes were chosen for each region under study, considering different pollutants: PM_10_ and O_3_.

**Figure 7:**
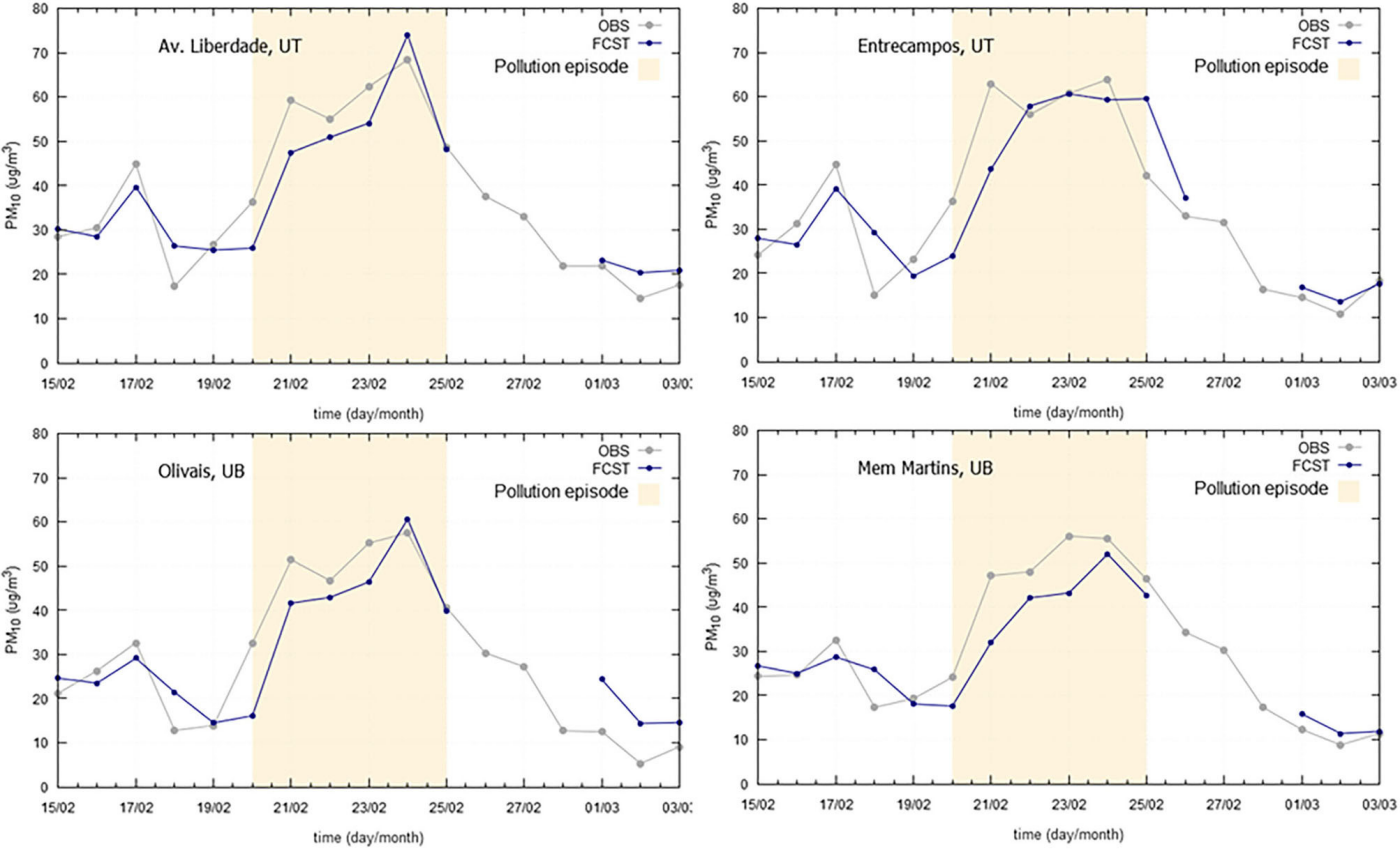
Particulate matter (PM_10_) observed (OBS) and forecasted (FCST) concentrations, with emphasis on the natural dust episode occurred in 2019 (20-25/02/2019), at four air quality monitoring stations at Greater Lisbon Area.

**Figure 8:**
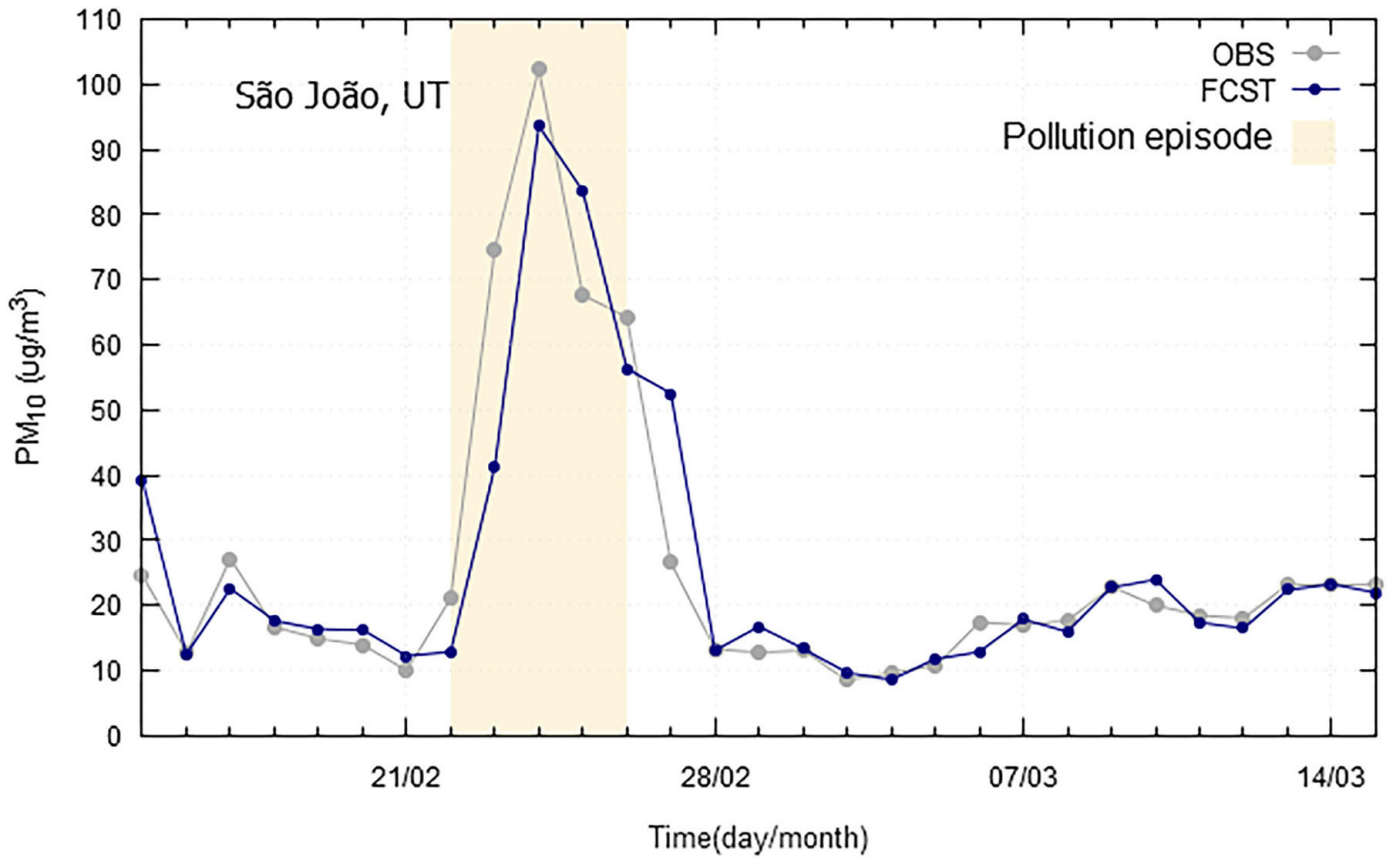
Particulate matter (PM_10_) observed (OBS) and forecasted (FCST) concentrations, with emphasis on the natural dust episode occurred in 2019 (22-26/02/2019), at São João air quality monitoring station at Madeira Autonomous Region.

Long-range transport processes of desert dust from North Africa are not infrequent, significantly affecting ground level particle concentrations recorded during these events, in Iberian Peninsula (Querol et al., 2009). In Portugal, both in Lisbon and Madeira, these natural dust intrusion episodes are common, contributing to higher PM_10_ concentrations, frequently above the daily limit value of 50 μg/m^3^, as represented in Figures 7, 8, often due to the persistence of specific synoptic patterns. Both in Lisbon and Madeira case studies, forecast models slightly underestimated PM_10_ concentrations, but were able to follow general PM_10_ evolution profile, showing a small delay in the prediction trend.

**Figure 9:**
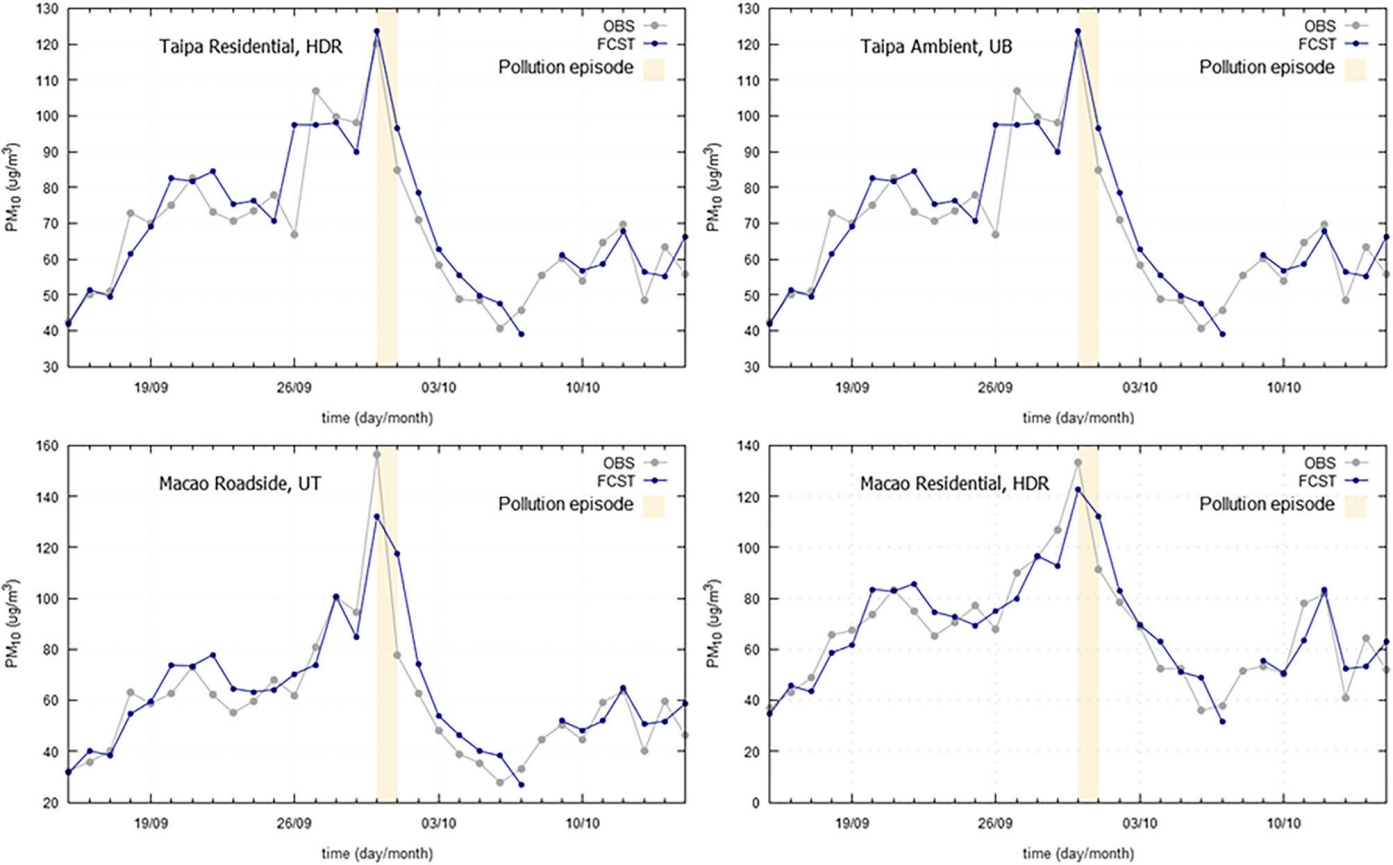
Particulate matter (PM_10_) observed (OBS) and forecasted (FCST) concentrations, with emphasis on the Chinese National Holiday in 2019 (01/10/2019), at four monitoring stations at Macao Administrative Region.

Concerning Macao Administrative Region, a period covering the Chinese National Holiday was chosen, in which a rise of PM_10_ concentrations, to values over 120 μg/m^3^, was measured at four air quality monitoring stations (Figure 9). This holiday is known to be a golden week of tourism, Macao being one of the favorite destinations for Chinese tourists (Lee et al., 2017) and also characterized by the release of a considerable amount of fireworks. The PM_10_ peak concentrations, occurred on the 1^st^ of October, was well predicted for Taipa monitoring locations and slightly underestimated for Macao stations.

**Figure 10:**
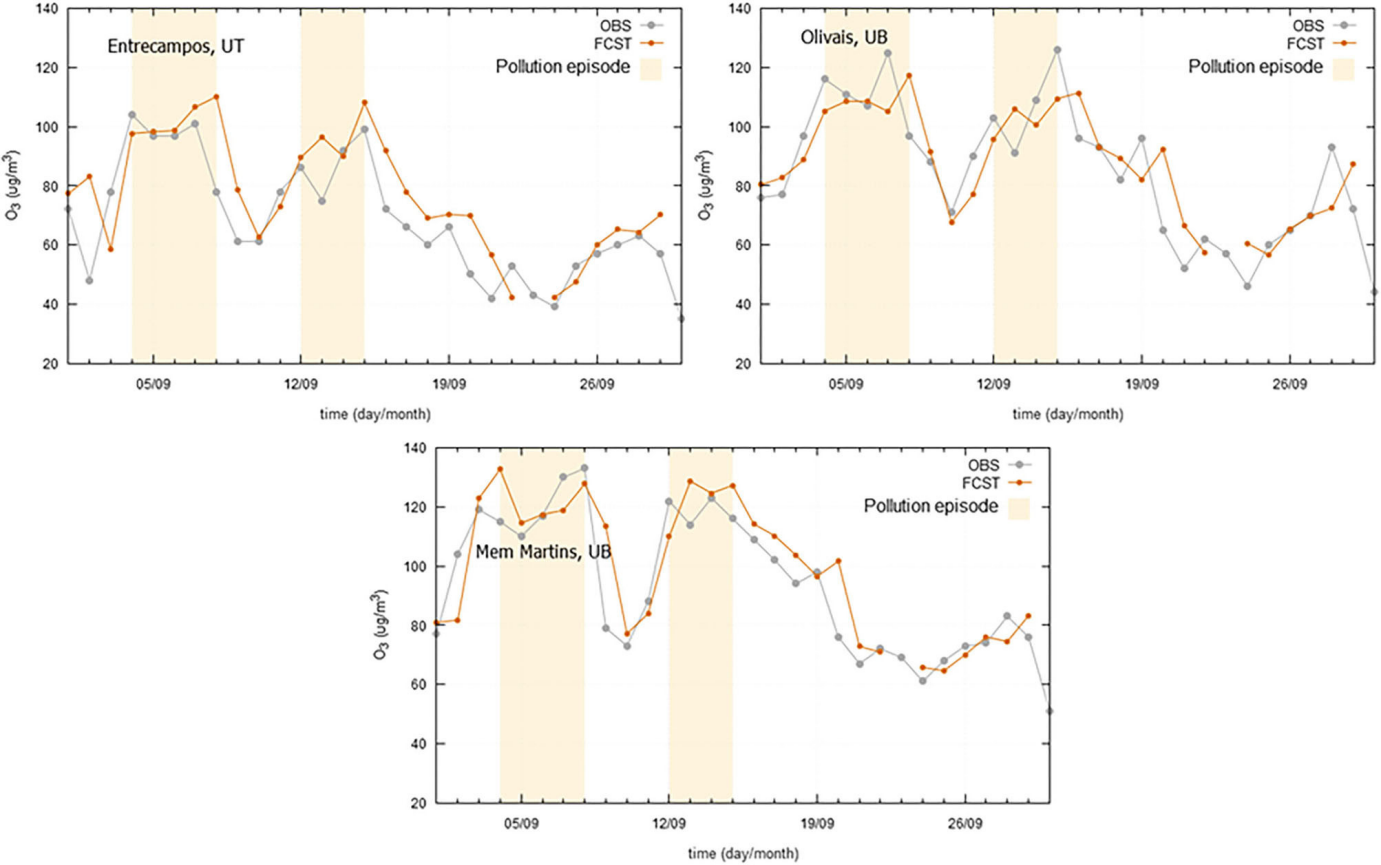
Ozone (O_3_) observed (OBS) and forecasted (FCST) concentrations, with emphasis on the pollution episodes occurred in 2019 (04-08/09/2019 and 12-15/09/2019), at three air quality monitoring stations at Greater Lisbon Area.

**Figure 11:**
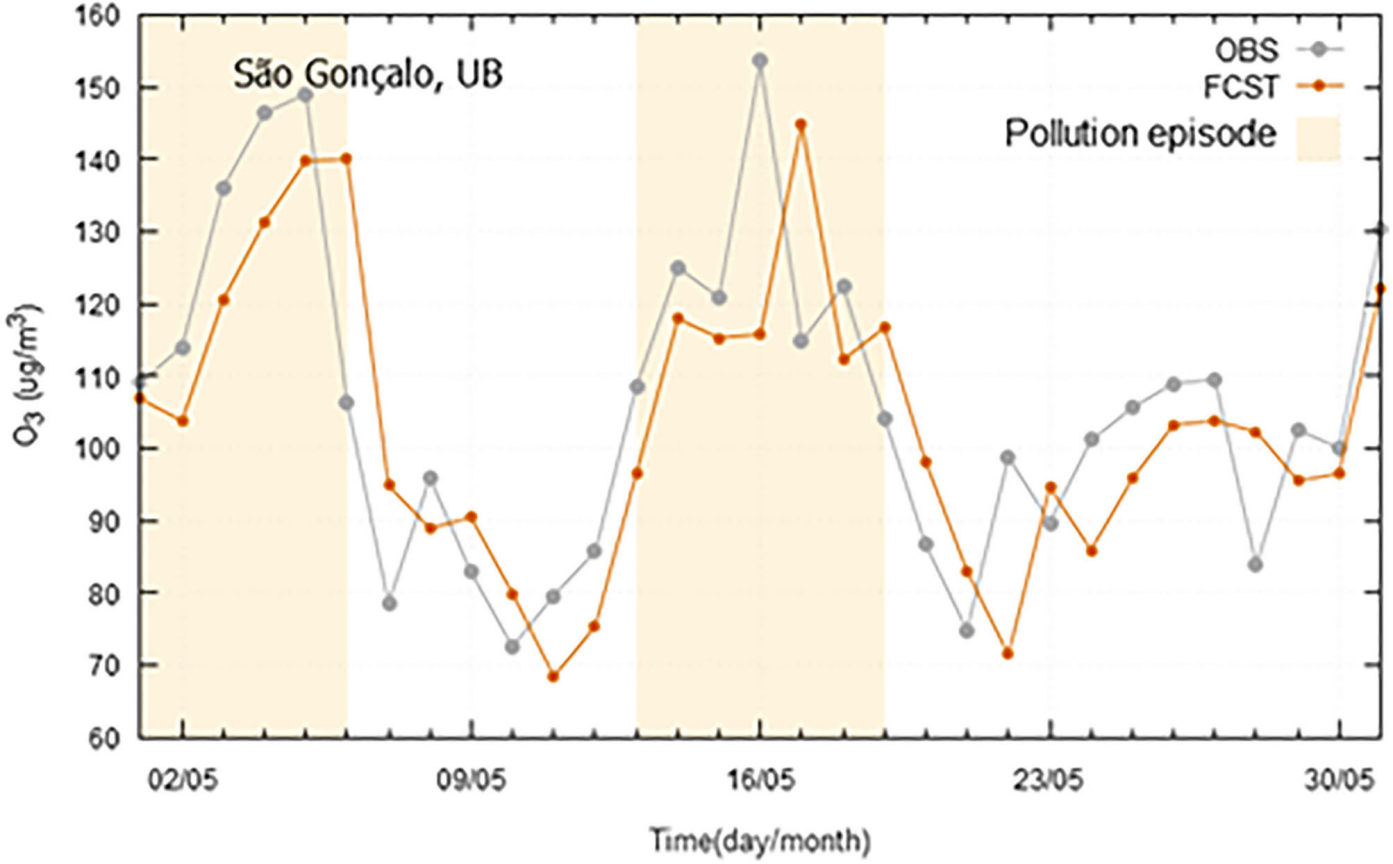
Ozone (O_3_) observed (OBS) and forecasted (FCST) concentrations, with emphasis on the pollution episode occurred in 2019 (01-06/05/2019 and 13-19/05/2019), at São Gonçalo air quality monitoring station at Madeira Autonomous Region.

**Figure 12:**
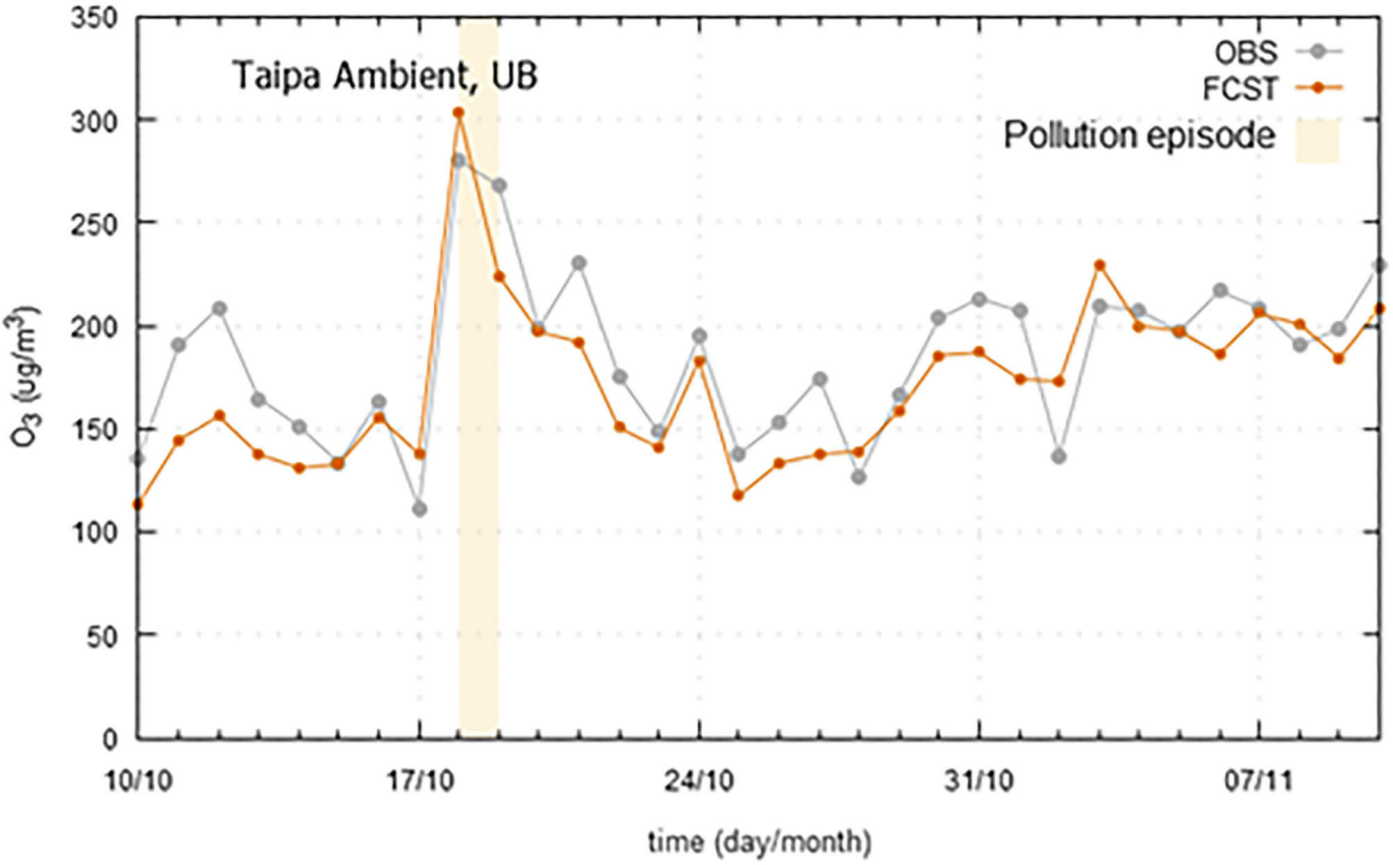
Ozone (O_3_) observed (OBS) and forecasted (FCST) concentrations, with emphasis on the pollution episode occurred in 2019 (18-19/10/2019), at Taipa Ambient air quality monitoring station at Macao Administrative Region.

Regarding O_3_, a set of pollution episodes, occurred in 2019 in the three case study regions, is presented in Figures 10–12. The marked high pollution intervals in these figures correspond to the exceedance of pollutant specific legal thresholds. Mechanisms for near-surface ozone formation and depletion are complex. Previous studies have shown that ozone production accelerates at high temperatures, which may be attributed not only to the temperature dependence of chemical reactions, but also to the weak winds which accompany high temperatures and heatwaves, and cause the atmosphere to stagnate and built up ozone levels (Pyrgou et al., 2018). In a general mode, all the models have shown a good agreement between the observed and forecasted concentrations and were able to forecast the pollution peaks with a good degree of precision. However, in the case of Madeira, due to the particular circumstances of being an island with extreme altitude variations, a meteorological next-day variable was not found to be integrated in the model and anticipate some higher ozone levels. Ozone, as secondary pollutant, has a complex formation process that creates higher forecast difficulties in certain geographical areas. Therefore, in some of these situations, the lag shown in Figure 11, between observed and predicted ozone concentrations, is mostly a consequence of the daily evolution trend from the day before.

## Discussion

The described statistical approach to air quality forecasting has proven to be successful, being able to forecast next day NO_2_, PM_10_, PM_2.5_, and O_3_ concentrations with a good performance reflected by the presented evaluation scores. The results differ slightly, between stations and pollutant, but overall the variables included in each model explain more than 90% of the variance of the independent variable in the development stage, value that usually decreases in the validation period. The application to different regions pretends to demonstrate the versatility of the methodology. It is expected a small degradation in the performance of the models when in operation, due to several factors, such as the uncertainty of meteorological forecasts.

Statistical models should be updated on a regular basis if there are, for instance, significant changes on local sources of air pollution, but can also be improved with the introduction of new variables, as predictors, in order to better explain part of pollutant variance.

The forecast models can show a slight delay in response to the short-term sudden variations on concentrations, once the previous day concentration is itself the independent variable considered as the best predictor since it explains most of the model variance. However, as shown in the selection of PM_10_ and O_3_ pollution episodes, this methodology was able to reproduce the trend and variations of monitored air pollutant concentrations. This shows that the regression models obtained can be reliably applied to forecast next-day pollutants concentrations across different magnitude levels of air pollution, being a useful tool for air pollution impacts mitigation.

The method has a few advantages when compared with numerical modeling, namely the lower complexity of development and implementation, and the fewer computing resources needed. On the disadvantages side, it can be pointed at the high dependence on a good operating air quality monitoring network and meteorological forecasts. In the case of Portugal, where next day forecasts provided by the Portuguese Environmental Agency are calculated by two methods (deterministic and stochastic) as part of an ensemble approach for both PM_10_ and O_3_, the quality data for 2019 show that the probability of detection, by the stochastic model was higher for all the regions (6) within the country except for one.

## Data Availability Statement

The presented datasets belong to public institutions. Requests to access these datasets should be directed to lc.mendes@fct.unl.pt.

## Author Contributions

LM, JM, and FF: data curation and writing—review and editing. FF: funding acquisition. LM: methodology, software, and writing—original draft. JM and FF: supervision. LM and JM: validation. All authors have read and agreed to the published version of the manuscript.

## Funding

This research is based on the outcomes from the Portuguese PrevQualar Project, supported by the Portuguese Environment Agency. Furthermore, the preparatory work performed for Macao Administrative Region was supported by the Macao's Meteorological and Geophysical Bureau (SMG). The research activities developed at CENSE are financed by the Portuguese Foundation for Science and Technology (FCT) through the Strategic Project UIDB/04085/2020. The presented work was made possible with the support by Portuguese Institute for Sea and Atmosphere (IPMA). Concerning the Macao modelling tasks, the work was supported by Dr. Man Tat-Lei.

## Conflict of Interest

The authors declare that the research was conducted in the absence of any commercial or financial relationships that could be construed as a potential conflict of interest.

## Publisher's Note

All claims expressed in this article are solely those of the authors and do not necessarily represent those of their affiliated organizations, or those of the publisher, the editors and the reviewers. Any product that may be evaluated in this article, or claim that may be made by its manufacturer, is not guaranteed or endorsed by the publisher.
